# Supplementary material for: Direct observation of heterogeneous formation of amyloid spherulites in real-time by super-resolution microscopy
Source: Commun Biol. 2022 Aug 20;5:850. doi: 10.1038/s42003-022-03810-1 (PMC9392779; doi:10.1038/s42003-022-03810-1)
Supplement: Supplementary file 2 — Supplementary Information [file 42003_2022_3810_MOESM2_ESM.pdf]

Supplementary Information

Direct Observation of Heterogeneous Formation of Amyloid Spherulites in Real-time by Super-resolution Microscopy

*Min Zhang, Henrik D. Pinholt, Xin Zhou, Søren S.-R. Bohr, Luca Banetta, Alessio Zaccone, Vito Foderà * and Nikos S. Hatzakis**

Supplementary Methods

Atto 655-labeled liposome preparation for 3D dSTORM calibration.

Atto 655-labeled liposomes with 2% negative charge which were used for the 3D dSTORM calibration were prepared as previously published method [1]. In detail, a ratio of 97/2/0.5/0.5 for 1,2-Dioleoyl-sn-Glycero-3-Phosphocholine (DOPC), 1,2-dioleoyl-sn-glycero-3-phospho-L-serine (sodium salt) (DOPS), 1,2-distearoyl-snglycero-3-phosphoethanolamine-N-[biotinyl(polyethylene glycol)-2000] (ammonium salt) DSPE-PEG$_{2000}$-biotin and Atto655-PE were added to a glass vial. The solvent chloroform was removed completely by nitrogen flow for about 10 minutes followed by vacuum for several hours. The lipid film was rehydrated in MES buffer (pH 5.6) to final total lipid concentration 0.5 mg/mL, vortexed for 30 seconds and incubated for 30 minutes. The sample was extruded 11 times through a polycarbonate membrane filter with a pore size 50 nm. Then the liposome suspension was exposed to 10 cycle of flash-freezing and thawing so as to ensure an unilamellar membrane structure. The liposomes were aliquoted and stored at -20 °C.

Supplementary Figures

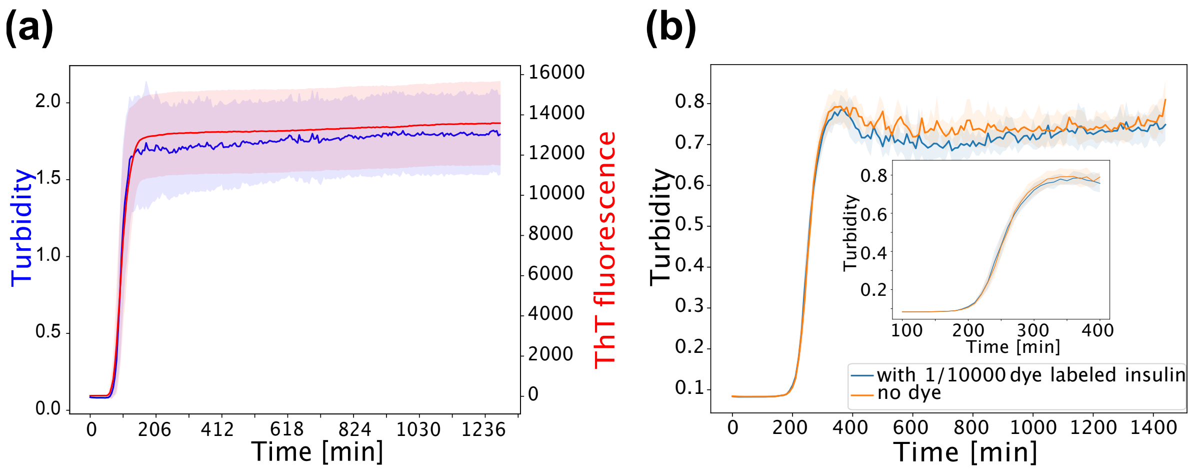

Supplementary Figure 1. a) ThT fluorescence and turbidity kinetics showing that the aggregation process was complete in ~ 3 hours at 60 °C and that turbidity and fluorescence provided identical results, supporting amyloid origin. b) Turbidity kinetics of human insulin at 45 °C with 1/10000 Alexa Fluor 647-labeled insulin (blue line) and without Alexa Fluor 647 (orange line). Each curve is the average on at least four replicates. The shaded lines present standard deviation. The consistent of turbidities confirms that labeling doesn’t interfere the aggregation process.

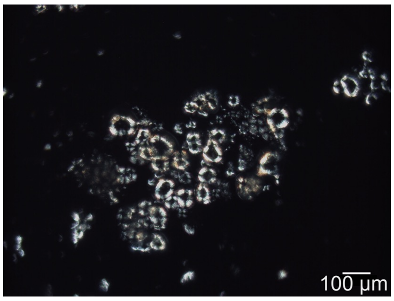

Supplementary Figure 2. Spherulites images obtained by cross polarised microscopy Zeiss Axioplan Optical Microscope, Carl Zeiss. The characteristic Maltese cross shows spherulite formation. Scale bar is 100 µm.

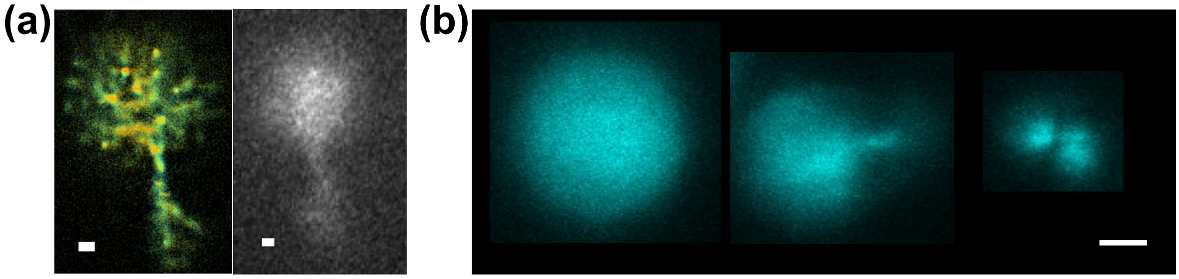

Supplementary Figure 3. Comparison of conventional microscopy with super resolution readouts here for spherulite structure identification. (a). Left: 3D dSTORM image of an anisotropically grown spherulite. Right: image of the same spherulite obtained by conventional microscopy where all structural features and dimension of the spherulite are averaged due to diffraction limit. (b). TIRF images of isotropic and anisotropic HI spherulites using conventional microscopy based on ThT chromophore. The spherulites in (b) were formed in a block heater at 45 C° for 24 h. Diffraction limited imaging masks the structural details of the aggregates that are visible in dSTORM and REPLOM. Note the images in (b) were recorded at very low laser power to avoid saturation. In these setting smaller and newly formed spherulites are not visible Scale bars: (a) 1 µm, (b) 5 µm.

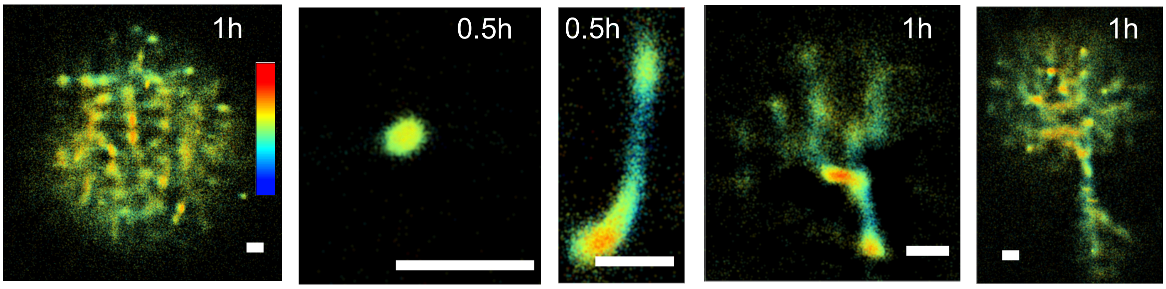

Supplementary Figure 4. 3D dSTORM images at different steps of the aggregate growth at incubation time from 0.5 hour to 1 hours and incubation temperature at 60 °C. Pseudocolor scale is from −500 nm to 500 nm; Scale bars are 1 μm.

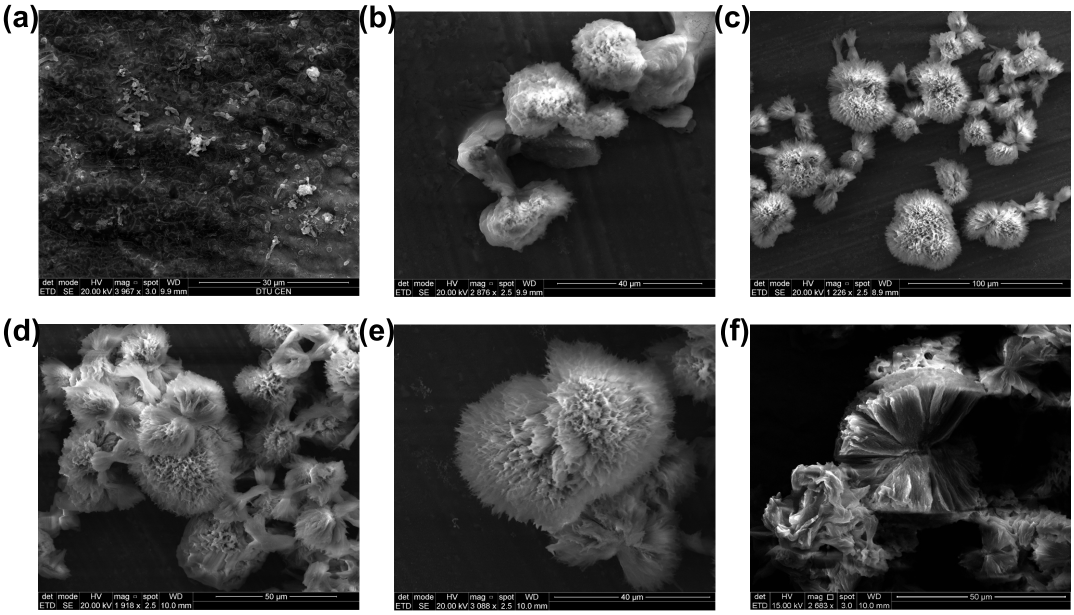

Supplementary Figure 5. SEM image of spherulites grew at 60 °C. (a) with incubation time of 1 hour in tube, (b) with incubation time of 2 hours in tube, (c) with incubation time of 4 hours and (d-e) with incubation time of 24 hours in tube. (e) a fully-grown insulin spherulite in tube, (f) with incubation time of 24 hours in microplate. Scale bars are (a) 30 μm, (b) 40 μm, (c) 100 μm, (d) 50 μm, (e) 40 μm and (f) 50 μm respectively.

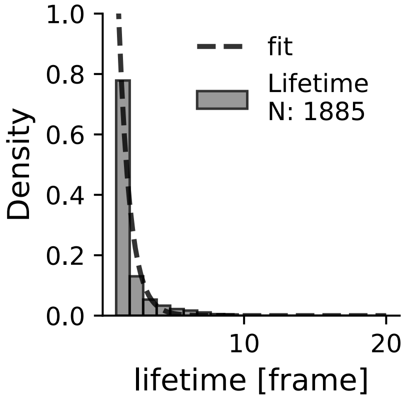

Supplementary Figure 6. Quantification of bleaching time of Alexa 647 in REPLOM conditions. In the experimental condition used (absence of imaging buffer and high laser power) Alexa 647 chromophores are rapidly photobleached. Lifetime is 0.7845 ± 0.0017 frames.

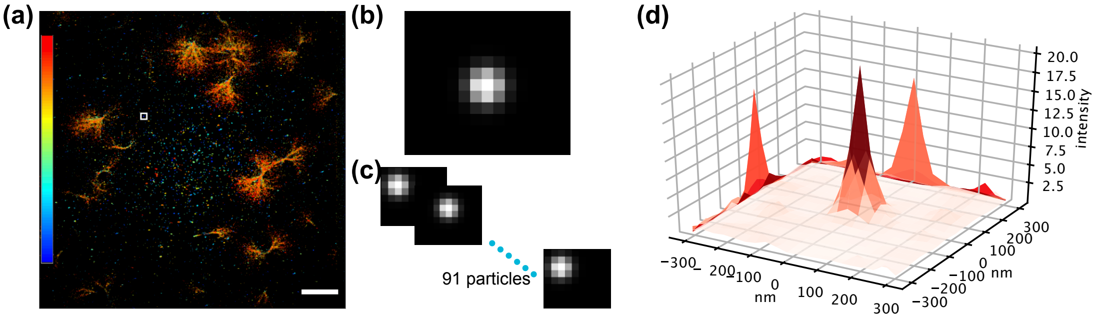

Supplementary Figure 7. Quantification of REPLOM resolution. (a) REPLOM image of HI spherulites grew at 45 °C. Scale bar: 10 µm. (b) Higher-magnification view of the boxed region in (a), which contains a single spot. (c) 91 particles were selected for resolution calculation. (d) Resulting 2D histograms were generated by aligning the 91 particles to the same center. Two-dimensional gaussian fitting to the presented histogram allowed extraction of FWHM for resolution determination. FWHM x: 68.1 nm, FWHM y: 66.2 nm.

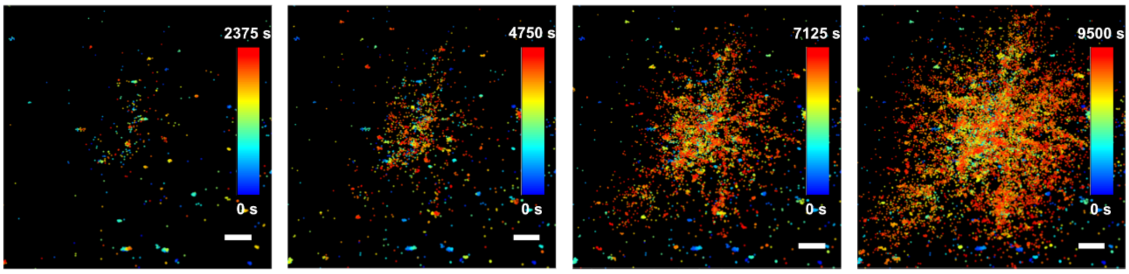

Supplementary Figure 8. Direct real-time observation of temporal development of isotropic growth at t = 2375 time intervals. Scale bars: 2 μm.

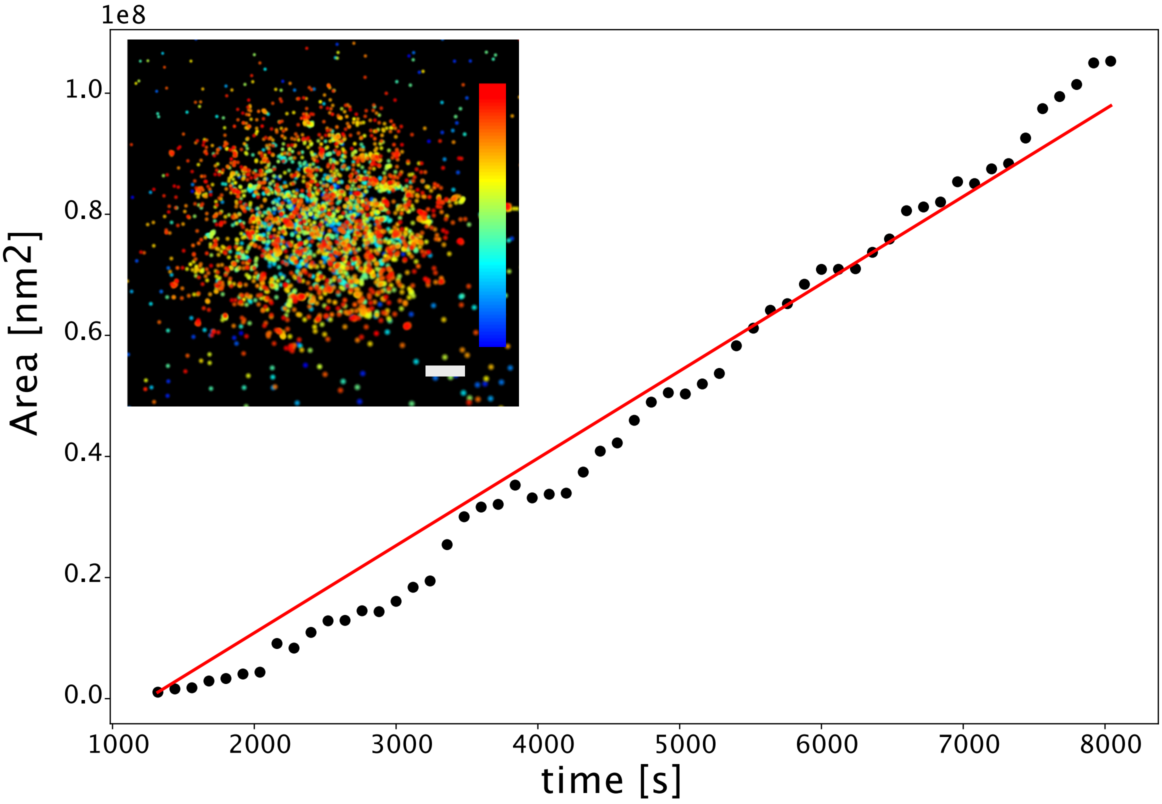

Supplementary Figure 9. Growth curve of an isotropic spherulite obtained by REPLOM with incubation temperature at 45 C° and temporal resolution of 2 minutes. The rate r_x is 14400 nm^2/s, which is consist with the REPLOM results with 25s temporal resolution. Scale bars: 2 μm. The pseudocolor bar represents time and spans from 0s to 8160s.

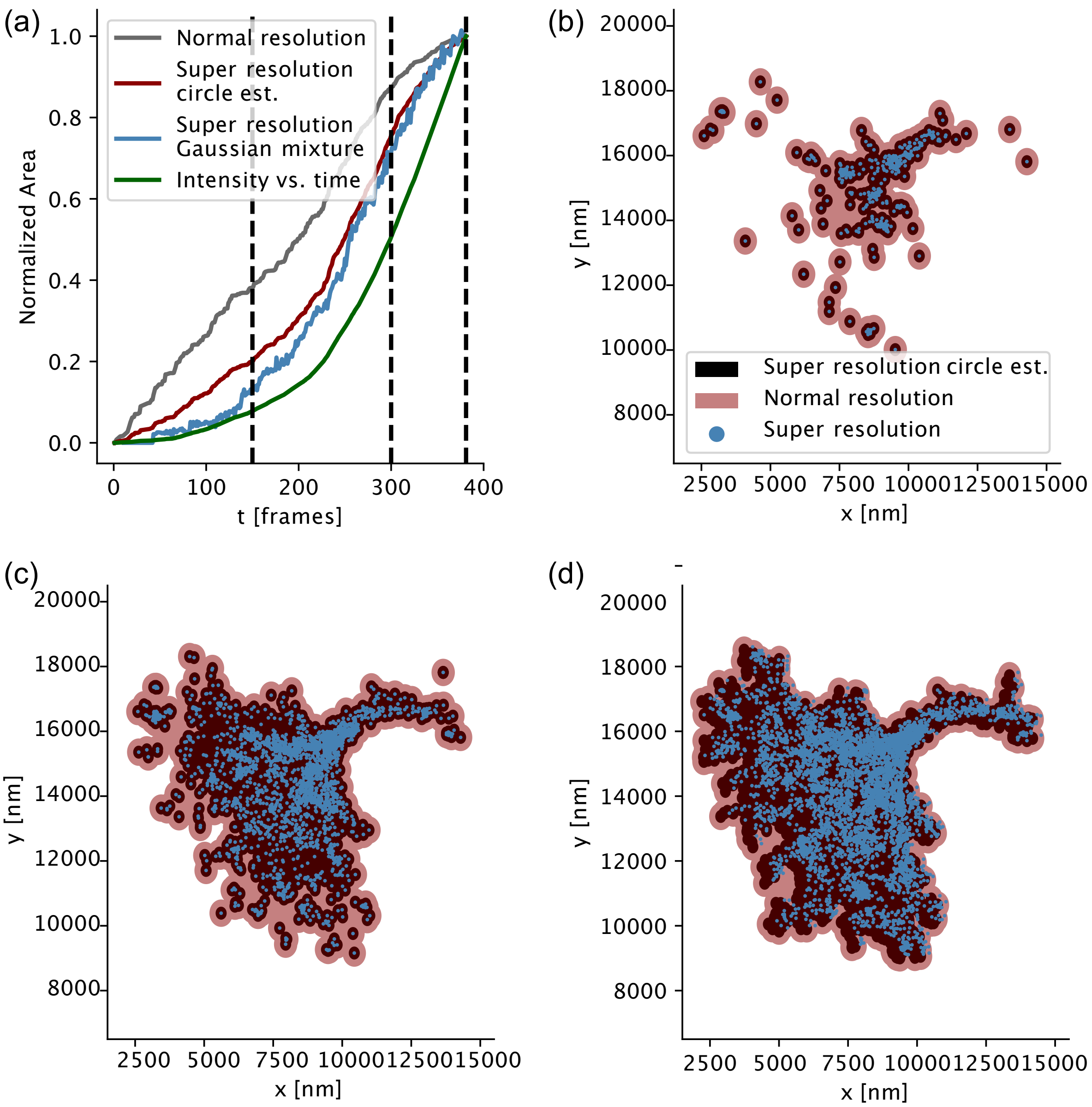

Supplementary Figure 10. Comparison of the effects of intensity counting vs. super resolution for area estimation in REPLOM. (a): Maximum-normalized estimated areas from the method used in the main text (blue curve), data from REPLOM with effects of all labeled monomers with infinite resolution (red curve, no PSF convolution), and the latter with the effects of a finite resolution included (grey curve). Note the lacking ability to resolve the two-state nature of the area growth with finite resolution. (b): Spatial plot of contributions to the area in the three methods for the first vertical line in a. (c): Same as b, second vertical line in a. (d): Same as b, third vertical line in d. The binding distribution with all monomers labeled was modeled as a uniform distribution centered on the spot from REPLOM with a width equal to that of the tail of the aggregate in the super resolution data (400 nm). The PSF width was taken to be lamda/(2*NA) (214.8 nm). Intensity vs. time was calculated as the cumulative count of REPLOM spots in time.

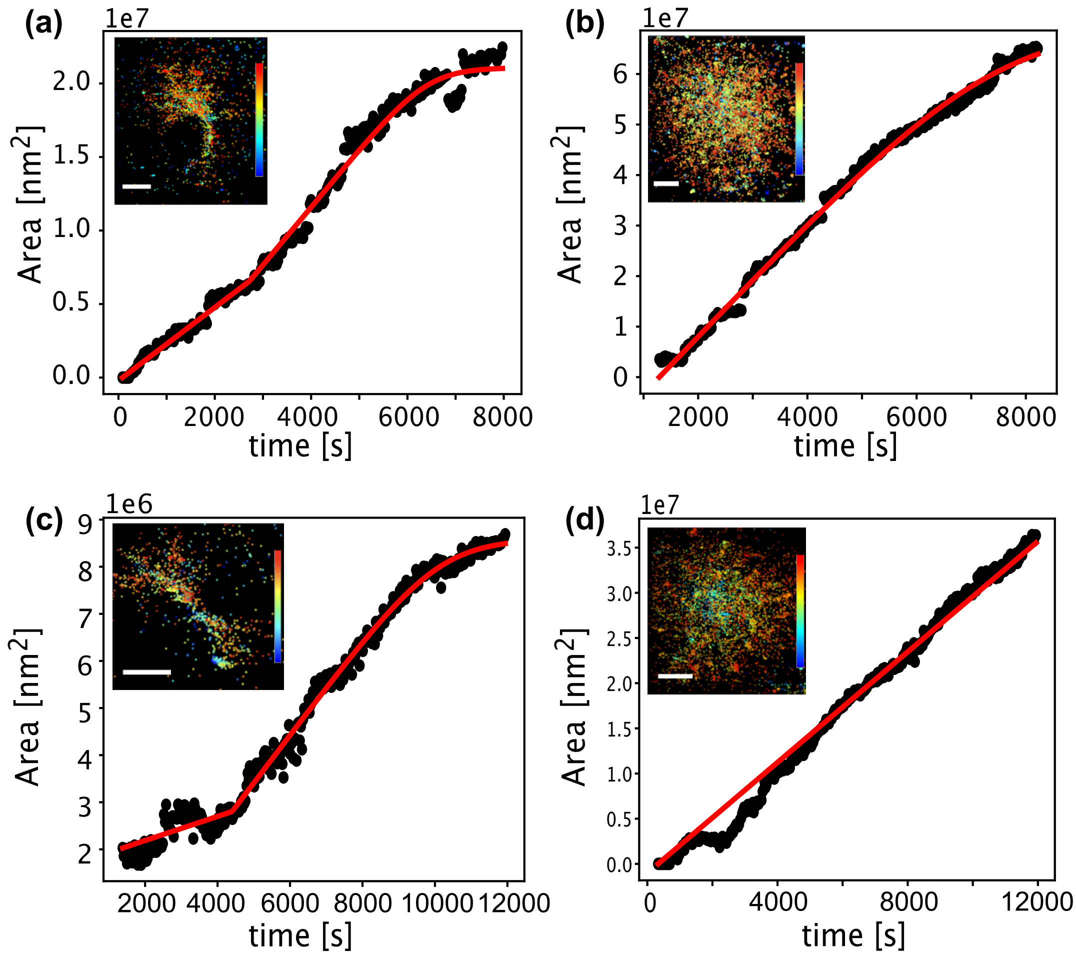

Supplementary Figure 11. Representative anisotropic (a, c) and isotropic (b, d) HI spherulites obtained by REPLOM and their corresponding growth curves. The incubation temperatures were: (a&b) 37 °C, and (c&d) 32 °C. Scale bars: 2 um. The pseudocolor bar represents time and spans from 0s to 8000s in (a and b) and from 0 s to 12000 s in (c and d).

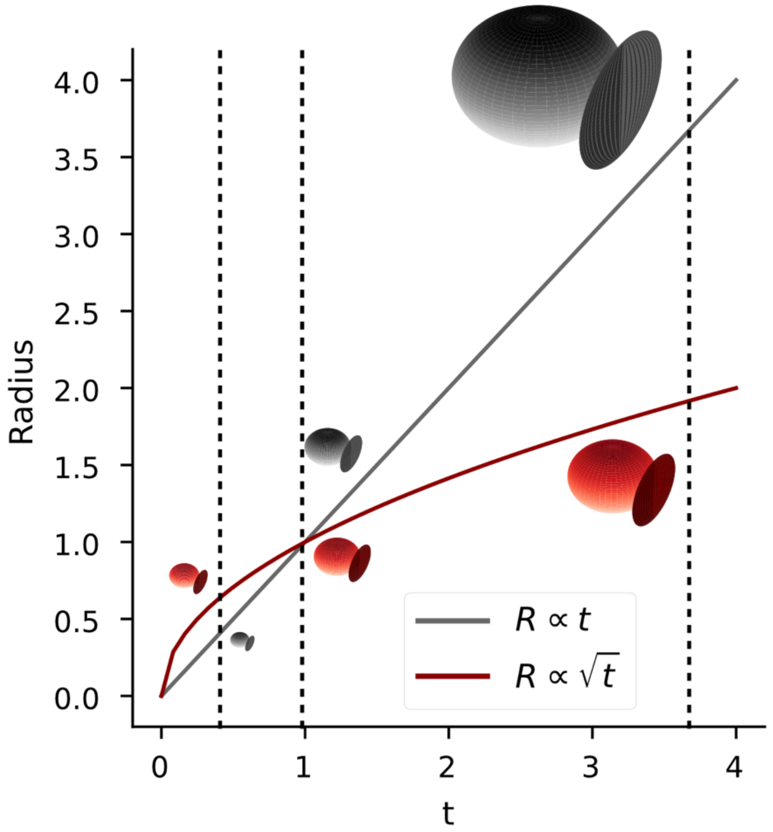

Supplementary Figure 12. Visualization of the two growth modalities used for fitting REPLOM data of insulin spherulites. Growth has been found to proceed either reaction limited or diffusion limited, leading to a linear extension which scales like $\sqrt{t}$ or t respectively. These dynamics were then directly translated into the dynamics of either the 2D projection or the full 3D volume through a simple differential equation (3D objects and associated projections, see methods).

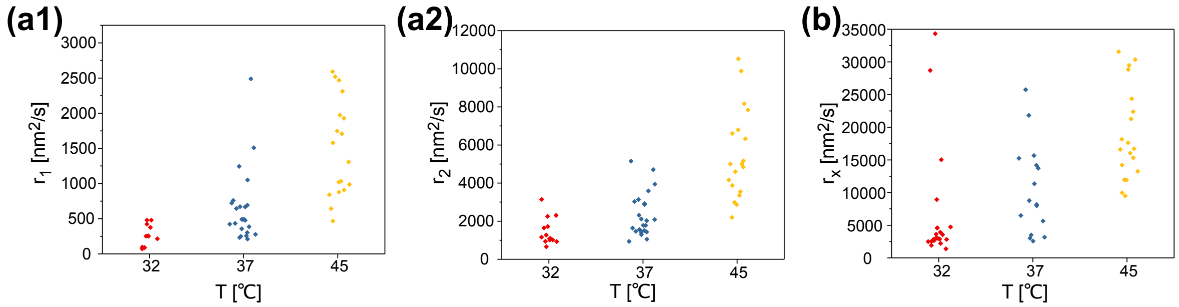

Supplementary Figure 13. Growth rate distribution for all tested temperatures (32, 37 and 45 °C) for all spherulite growth morphologies. a1) for r_1 of anisotropic growth a2) for r_2 of anisotropic growth b) r_x for isotropic growth. Data extracted from Arrhenius plots in Figure 4.

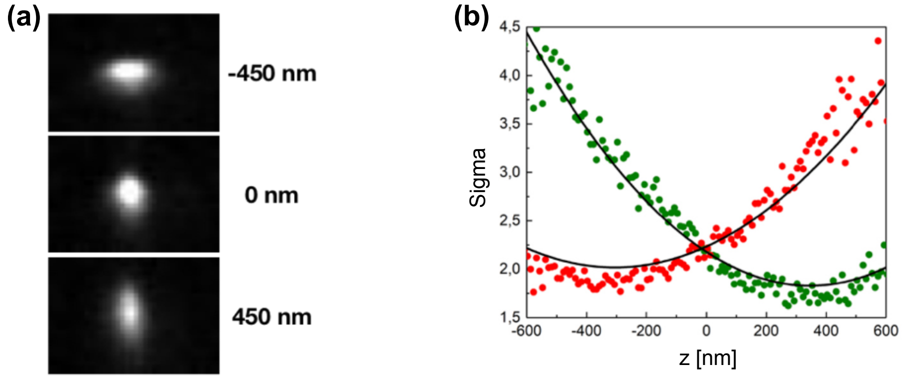

Supplementary Figure 14. (a) Single localization imaging of Atto-655 labeled liposomes extruded at 50nm and tethered on poly-L-Lysine passivated surfaces. (b) Calibration curve of PSF widths as a function of z. Z step length was 10 nm. The calibration curve was calculated by ThunderSTORM.

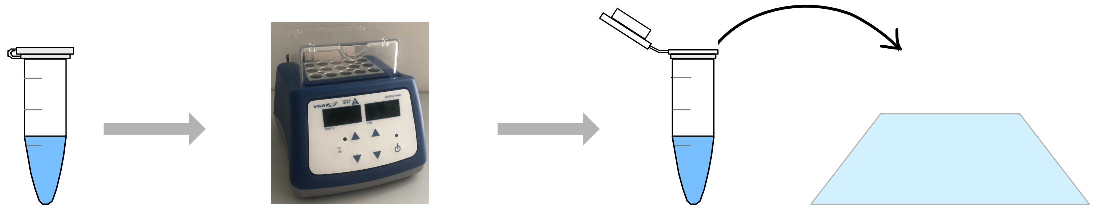

Supplementary Figure 15. All the samples were pre-incubated in a block heater in the designated temperature to skip the lag-phase. Preincubated samples were then put on a microscope slide to monitor spherulite growth on the microscope.

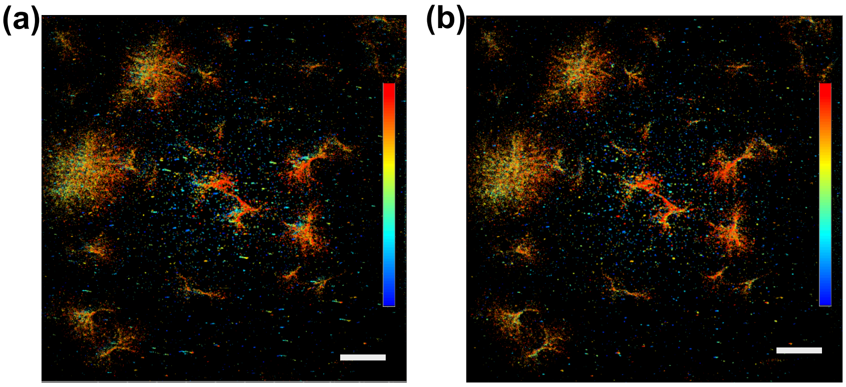

Supplementary Figure 16. REPLOM image of HI spherulites grown at 45 °C (a) prior to and (b) after drift correction. Scale bars: 10 µm. The pseudocolor represents time and ranges from 0 s (blue) to 9500 s (red). It worth noticing that if spherulites initiate their growing during the late of the recording, there will be no blue/green pseudocolor and most of the structure will be in red pseudocolor.

Supplementary Table 1. Quantification of the diameters of the central fibrils of the intermediates in Figure 1.

**Intermediates in Figure 1**	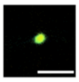	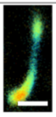	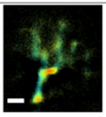	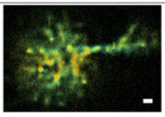
**Diameter of the central fibrils (nm)**	~300	~400	~450	~700

Supplementary Table 2. The relative abundance of the types of spherulites with different incubation temperatures.

	45°C	37°C	32°C
Isotropic	25 (33%)	37 (32%)	44 (45%)
Anisotropic	47 (62%)	73 (63%)	44 (45%)
Linear	4 (5%)	6 (5%)	9 (9%)

Supplementary References

1 Thomsen, R. P. *et al.* A large size-selective DNA nanopore with sensing applications. *Nat. Commun.* **10**, 5655, doi:10.1038/s41467-019-13284-1 (2019).
