# Supplementary material for: Direct observation of heterogeneous formation of amyloid spherulites in real-time by super-resolution microscopy
Source: Commun Biol. 2022 Aug 20;5:850. doi: 10.1038/s42003-022-03810-1 (PMC9392779; doi:10.1038/s42003-022-03810-1)
Supplement: Supplementary file 3 — Description of Additional Supplementary Files [file 42003_2022_3810_MOESM3_ESM.pdf]

Description of Additional Supplementary Files

File name: Supplementary Movie 1
Description: 3D rotation video of anisotropic spherulite with asymmetric lobes.

File name: Supplementary Movie 2
Description: 3D rotation video of anisotropic spherulite with symmetric lobes

File name: Supplementary Movie 3.

Description: Real-time growth and the corresponding area of the anisotropic spherulite in Figure 3c (incubation temperature 45°C, exposure time of 30 ms followed by a waiting time for each frame of 25 s)

File name: Supplementary Movie 4.

Description: Real-time growth and the corresponding area of the isotropic spherulite in Figure 3d (incubation temperature 45°C, exposure time of 30 ms followed by a waiting time for each frame of 25 s)

File name: Supplementary Movie 5.

Description: Real-time growth and the corresponding area of the isotropic spherulite in Supplementary Figure 9 (incubation temperature 45°C, exposure time of 30 ms followed by a waiting time for each frame of 120 s)

File name: Supplementary Movie 6.

Description: Real-time growth video of HI spherulite obtained by conventional TIRF microscope with 3 µM ThT as the chromophore (incubation temperature 45°C, exposure time of 30 ms followed by a waiting time for each frame of 25 s)

File name: Supplementary Movie 7.

Description: Real-time growth and the corresponding area of the anisotropic spherulite in Supplementary Figure 11a (incubation temperature 37°C, exposure time of 30 ms followed by a waiting time for each frame of 27.4 s).

File name: Supplementary Movie 8

Description: Real-time growth and the corresponding area of the isotropic spherulite in Supplementary Figure 11b (incubation temperature 37°C, exposure time of 30 ms followed by a waiting time for each frame of 27.4 s)

File name: Supplementary Movie 9.

Description: Real-time growth and the corresponding area of the anisotropic spherulite in Supplementary Figure 11c (incubation temperature 32°C, exposure time of 30 ms followed by a waiting time for each frame of 30 s)

File name: Supplementary Movie 10.

Description: Real-time growth and the corresponding area of the isotropic spherulite in Supplementary Figure 11d (incubation temperature 37°C, exposure time of 30 ms followed by a waiting time for each frame of 30 s)

File name: Supplementary Movie 11.

Description: Atto655 labeled insulin hexamers were immobile on the surface without molecular diffusion (exposure time of 30 ms followed by a waiting time for each frame of 25s)
