# Supplementary material for: Direct observation of heterogeneous formation of amyloid spherulites in real-time by super-resolution microscopy
Source: Commun Biol. 2022 Aug 20;5:850. doi: 10.1038/s42003-022-03810-1 (PMC9392779; doi:10.1038/s42003-022-03810-1)
Supplement: Supplementary file 15 — Reporting Summary [file 42003_2022_3810_MOESM15_ESM.pdf]

nature research

Corresponding author(s): Vito Foderà, Nikos S. Hatzakis

Last updated by author(s): Jul 4, 2022

Reporting Summary

Nature Research wishes to improve the reproducibility of the work that we publish. This form provides structure for consistency and transparency in reporting. For further information on Nature Research policies, see our Editorial Policies and the Editorial Policy Checklist.

Statistics

For all statistical analyses, confirm that the following items are present in the figure legend, table legend, main text, or Methods section.

n/a	Confirmed	
☐	☒	The exact sample size (n) for each experimental group/condition, given as a discrete number and unit of measurement
☐	☒	A statement on whether measurements were taken from distinct samples or whether the same sample was measured repeatedly
☒	☐	The statistical test(s) used AND whether they are one- or two-sided<br>*Only common tests should be described solely by name; describe more complex techniques in the Methods section.*
☐	☒	A description of all covariates tested
☒	☐	A description of any assumptions or corrections, such as tests of normality and adjustment for multiple comparisons
☐	☒	A full description of the statistical parameters including central tendency (e.g. means) or other basic estimates (e.g. regression coefficient) AND variation (e.g. standard deviation) or associated estimates of uncertainty (e.g. confidence intervals)
☒	☐	For null hypothesis testing, the test statistic (e.g. F, t, r) with confidence intervals, effect sizes, degrees of freedom and P value noted<br>*Give P values as exact values whenever suitable.*
☐	☒	For Bayesian analysis, information on the choice of priors and Markov chain Monte Carlo settings
☐	☒	For hierarchical and complex designs, identification of the appropriate level for tests and full reporting of outcomes
☒	☐	Estimates of effect sizes (e.g. Cohen's d, Pearson's r), indicating how they were calculated

Our web collection on statistics for biologists contains articles on many of the points above.

Software and code

Policy information about availability of computer code

Data collection	The data obtained by fluorescent microscopes, including spinning disk microscopy, 3D dSTORM and REPLOM, was collected by cellSens software (Olympus).
Data analysis	The 3D dSTORM and REPLOM data was analyzied by ThunderSTORM to get the reconstructed super-resolution images. Then the REPLOM data was further analyzied by a home-made software worked in python, which can be found in our GitHub.

For manuscripts utilizing custom algorithms or software that are central to the research but not yet described in published literature, software must be made available to editors and reviewers. We strongly encourage code deposition in a community repository (e.g. GitHub). See the Nature Research guidelines for submitting code & software for further information.

Data

Policy information about availability of data

All manuscripts must include a data availability statement. This statement should provide the following information, where applicable:

- Accession codes, unique identifiers, or web links for publicly available datasets
- A list of figures that have associated raw data
- A description of any restrictions on data availability

All data sets used for figures are provided as source data in the manuscript. The source data are available at https://sid.erda.dk/sharelink/fje3exOlq2.

Field-specific reporting

Please select the one below that is the best fit for your research. If you are not sure, read the appropriate sections before making your selection.

☒ Life sciences ☐ Behavioural & social sciences ☐ Ecological, evolutionary & environmental sciences

For a reference copy of the document with all sections, see nature.com/documents/nr-reporting-summary-flat.pdf

Life sciences study design

All studies must disclose on these points even when the disclosure is negative.

Sample size	Our study was about the growth mechanism of insulin spherulites. So we didn't perform sample-size calculation. We just made sure our sample sizes were sufficient to support our findings.
Data exclusions	If two spherulites were too close to separate them, the software may consider they belong to one spherulite. We excluded this kind of data when statistics the growth rates of individual spherulites.
Replication	Most of the measurements were taken at least three times, and the experimental findings were reproduced successfully.
Randomization	When performing REPLOM, we randomly selected several areas on the glass slides and started recording.
Blinding	Blinding was not relevant to our study, when we collected the data, we knew all the experimental conditions.

Reporting for specific materials, systems and methods

We require information from authors about some types of materials, experimental systems and methods used in many studies. Here, indicate whether each material, system or method listed is relevant to your study. If you are not sure if a list item applies to your research, read the appropriate section before selecting a response.

Materials & experimental systems

n/a	Involved in the study	
☒	☐	Antibodies
☒	☐	Eukaryotic cell lines
☒	☐	Palaeontology and archaeology
☒	☐	Animals and other organisms
☒	☐	Human research participants
☒	☐	Clinical data
☒	☐	Dual use research of concern

Methods

n/a	Involved in the study	
☒	☐	ChIP-seq
☒	☐	Flow cytometry
☒	☐	MRI-based neuroimaging
